# Supplementary material for: Enablers and barriers to the implementation of socially assistive humanoid robots in health and social care: a systematic review
Source: BMJ Open. 2020 Jan 9;10(1):e033096. doi: 10.1136/bmjopen-2019-033096 (PMC6955545; doi:10.1136/bmjopen-2019-033096)
Supplement: Supplementary data [file bmjopen-2019-033096supp001.pdf]

DATA EXTRACTION TABLE

Name of study: **Assessor**

**Interventions: dose/frequency/duration etc**
**Intervention**<br><br>**Comparator**

**Outcome measures according to us, of our review (not from authors of study)**			
1. **Primary outcomes**<br>The identification of a comprehensive listing of enablers and barriers to the implementation of Socially Assistive Humanoid Robots (SAHR) in health and social care.<br>Barriers: those impeding the implementation of SAHR which may include factors, issues or themes at local, system or policy level.<br>Enablers: mechanisms and initiatives whereby patients' providers or policy makers contribute to facilitating the positive uptake and implementation of SAHR.<br>2. **Secondary outcomes**<br>The insights from these enablers and barriers and their impact.<br>Gaps and future developments to inform further research.<br><br>**Principal outcome measures: (from the authors in the study)**<br>a) ...<br>b) …<br>c) …			
**What were the results of these outcome measures?**			
**Measure a)**			
	Pre	Post	
1.			Fisher's exact
2.			
**Measure b)** and **c)**			
**Barriers**			
**Enablers**			
**Gaps and future developments**			

**SUMMARY**	
Identified Enablers from this study:	1. …
	2. …
Identified Barriers:	1. …
	2. …
**Conclusive remarks**	
**Useful References**	
